# Defining the impact of adjuvant treatment on the prognosis of patients with inoperable glioblastoma undergoing biopsy only: does the survival benefit outweigh the treatment effort?

**DOI:** 10.1007/s10143-022-01754-y

**Published:** 2022-02-23

**Authors:** Ronja Löber-Handwerker, Katja Döring, Christoph Bock, Veit Rohde, Vesna Malinova

**Affiliations:** Department of Neurosurgery, University Medical Center Göttingen, Robert-Koch-Str. 40, 37075 Göttingen, Germany

**Keywords:** Glioblastoma, Radio-chemotherapy, Overall survival, Prognostic factors, Inoperable glioblastoma

## Abstract

Patients with inoperable glioblastoma (GBM) usually experience worse prognosis compared to those in whom gross total resection (GTR) is achievable. Considering the treatment duration and its side effects identification of patients with survival benefit from treatment is essential to guarantee the best achievable quality of life. The aim of this study was to evaluate the survival benefit from radio-chemotherapy and to identify clinical, molecular, and imaging parameters associated with better outcome in patients with biopsied GBMs. Consecutive patients with inoperable GBM who underwent tumor biopsy at our department from 2005 to 2019 were retrospectively analyzed. All patients had histologically confirmed GBM and were followed up until death. The overall survival (OS) was calculated from date of diagnosis to date of death. Clinical, radiological, and molecular predictors of OS were evaluated. A total of 95 patients with biopsied primary GBM were enrolled in the study. The mean age was 64.3 ± 13.2 years; 56.8% (54/95) were male, and 43.2% (41/95) female. Median OS in the entire cohort was 5.5 months. After stratification for adjuvant treatment, a higher median OS was found in the group with adjuvant treatment (7 months, range 2–88) compared to the group without treatment (1 month, range 1–5) log-rank test, *p* < 0.0001. Patients with inoperable GBM undergoing biopsy indeed experience a very limited OS. Adjuvant treatment is associated with significantly longer OS compared to patients not receiving treatment and should be considered, especially in younger patients with good clinical condition at presentation.

## Introduction

Glioblastoma (GBM) is associated with very limited survival odds requiring a well-balanced management between aggressive treatment to increase the chance of longer survival and the preservation of quality of life. The median overall survival (OS) of GBM patients is 15 months, whereas only 3–5% survive longer than 3 years, who are then already considered long-term survivors. The 5-year survival rate was 6.8% [15]. The 10-year survival rate with GBM was estimated to be 0.71% [22]. Besides younger age and good clinical status at diagnosis with independency during everyday life activities, the extent of resection (EOR) is considered a clinically relevant prognostic factor in GBM patients [10]. Since application of 5-aminolevulin acid (ALA) has significantly improved the rates of gross total resection (GTR) resulting in increased overall (OS) and progression free survival (PFS), GTR whenever achievable has been pursued as a surgical goal during glioma surgery [19]. However, the risk of postoperative neurological deficits increases with radical tumor resection outlining a delicate line between reaching GTR and avoiding neurological deficits. Especially in eloquently located tumors, GTR is often not feasible, even by means of intraoperative neuromonitoring tools [9]. Since neurological deficits are directly associated with worse outcome, the benefit of more radical tumor resection at the expense of neurological deficits is questionable. Considering the increasing knowledge about GBM growth patterns as widespread infiltrating tumor at the time of diagnosis, GTR does not seem to be a reasonable surgical goal in patients with multifocal or eloquent GBM [12, 16, 21]. Hence, most neurooncological centers only perform a tumor biopsy for histological confirmation and treatment planning in these patients. The patient population with inoperable GBM is expected to have a very limited survival since they cannot receive maximal available treatment. The actual survival benefit of the radio-chemotherapy in this patient population has not been determined yet. Considering the limited prognosis of these patients on the one side and the strain of adjuvant treatment on the other side, it is of great clinical interest to evaluate the impact of radio-chemotherapy on survival in this specific patient population. The primary objective of this study was to identify factors associated with longer survival in a patient population with newly diagnosed inoperable GBM with or without subsequent tumor-specific treatment after histological diagnosis confirmation by biopsy-only. The study goals were as follows: (1) to shed light on this heterogenous patient population with solely biopsied GBM and to elucidate the criteria for inoperability, (2) to assess whether the survival benefit from tumor treatment overweighs the associated burden of radio-chemotherapy regarding the very limited life expectancy, (3) to identify radiological and molecular tumor-specific factors alongside with patient-specific parameters associated with longer OS in patients with biopsied GBM considered inoperable.

## Methods

### Patient population

**Fig. 1:**
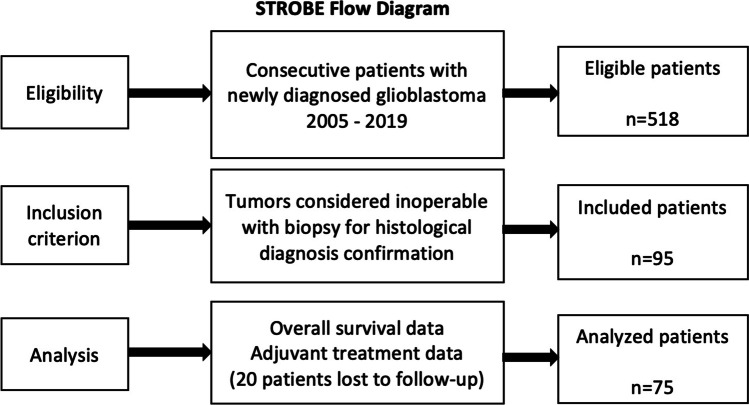
STROBE flow diagram demonstrating the definition of the study population

We performed a retrospective analysis of a consecutive and homogenous patient cohort treated in the time period after the introduction of the Stupp protocol in 2005. GBM was histologically confirmed after performing a biopsy of tumors considered inoperable. The reasons for considering the tumor as inoperable were extracted from medical records and operations reports. To maintain comparable standards of treatment, patients treated at our department in the time interval from 2005 to 2019 were included in the analysis of the study (Fig. 1). Patient-specific parameters like age at diagnosis, Karnofsky Performance Status (KPS) at presentation, tumor-specific parameters including tumor location and distribution on imaging, and molecular markers such as the presence of MGMT (O-6-methylguanine-DNA methyltransferase) promoter methylation and IDH (isocitrate dehydrogenase) mutation were documented. Additionally, data regarding adjuvant treatment and treatment response as seen on imaging during the follow-up examinations were gathered. Treatment decisions were made after interdisciplinary case discussion by the institutional tumor board for tumors of the central nervous system. Volumetric analysis of the tumor on initial magnetic resonance imaging (MRI) was performed using the Brainlab software Elements (Brainlab® Munich, Germany).

### Outcome parameters

OS was defined as the time in months from diagnosis to the date of death. Data on patients who were lost to follow-up were censored. The impact of patient-specific, radiological, and molecular tumor-specific markers on survival was evaluated. The treatment response on imaging was analyzed according to the response assessment in neuro-oncology criteria (RANO), at 3- and 6-month follow-up imaging. PFS was defined as time from diagnosis to tumor progression on follow-up imaging according to the RANO criteria for tumor progression [25].

### Statistical analysis

The statistical analyses were performed by means of GraphPad Prism (Version 9, GraphPad Software, San Diego, CA, USA). For the presentation of baseline data, descriptive statistics, and frequency, distribution analysis was done. Continuous variables are depicted as means ± standard deviation (SD), categorical variables as frequencies or percentages. To analyze the difference of continuous variables and categorical data, *t*- and chi-squared tests were applied respectively. By performing univariate and multivariate linear regression models, we analyzed the effects of patient/tumor characteristics on OS. Statistical significance was set as *p* < 0.05. Overall survival rates were calculated by the Kaplan–Meier method applying a log-rank test.

## Results

### Patient characteristics

A total of 518 patients with newly diagnosed GBM were treated at our department between 2005 and 2019 (15 years), of whom 95 patients (18.3%) received a tumor biopsy without subsequent resection and were eligible for inclusion into further analysis of this study. Mean age was 64.3 ± 13.2 (range 29 to 88) years, 56.8% (54/95) were male, and 43.2% (41/95) were female. The median KPS at admission was 70% (range 40–100). Baseline characteristics are depicted in Table 1. The mean age of patients who received adjuvant treatment was significantly lower compared to the patient group without tumor treatment, while the other baseline characteristics were not significantly different.

**Table 1:** Characteristics of A patients with biopsied glioblastoma; B biopsied tumor within our studied patient cohort

Variable		All (*n* = 95)	Tumor therapy (*n* = 63)	No therapy (*n* = 12)	*p-*value
**A Baseline patient characteristics**					
Age in years	Mean (SD) Median (range)	64.3 (13.2) 65 (29–88)	61.9 (11.8) 64 (30–83)	73.9 (9.5) 75.5 (56–84)	0.001**
Sex	Male Female	56.8% 43.2%	63.5% 36.5%	41.7% 58.3%	0.16
KPS %	Mean (SD) Median (range)	72.9 (14) 70 (40–100)	73.7 (11.9) 70 (50–100)	69.2 (22.3) 70 (40–100)	0.65
**B Tumor characteristics**					
Tumor volume	Mean (SD) Median (range)	28.3 (20.8) 21.5 (5–97)	26.3 (20.8) 18 (5–97)	37.8 (20) 30 (20–68)	0.12
Tumor location	Corpus callosum Basal ganglia Motor cortex Multifocal	54.1% 31.1% 18% 16.4%	51.8% 33.9% 19.6% 17.9%	80% 20% 0% 0%	0.10 0.12 0.28 0.30
Molecular markers	MGMT methylation IDH mutation	34.4% 6.7%	33.3% 7.1%	50% 0%	0.64 0.70

### Tumor characteristics

The decision-making criteria whether a tumor was considered inoperable were based on its localization and distribution on initial imaging. Main reasons for a biopsy-only approach were expected neurological deficits by performing GTR. For example, in tumors involving eloquent brain structure or the corpus callosum (bilateral infiltration = butterfly GBM), basal ganglia, brain stem, or primary motor cortex, GTR was not reasonably achievable; hence, biopsy-only was performed. Multifocal tumor growth was another common criterion for biopsy-only instead of a resection of all tumor loci. Tumor volume was calculated in 80/95 (84.2%) patients. The initial imaging of 15 patients between 2005 and 2007 was not available digitally, impeding volumetric analysis. Mean initial tumor volume was 28.2 ± 21.2 ml and median 23.5 ml (range 5–100 ml). In 9.5% (9/95), tumors demonstrated multifocal/-local tumor growth, 23.2% (22/95) involved the corpus callosum, and 45.3% (43/95) had a contact to the ventricle system (Table 1). MGMT promoter methylation was found in 34.4% of the patient population. An IDH mutation was detected in 6.7% of all patients (Table 1). There were no statistically significant differences regarding tumor characteristics between the two patient groups (Table 1).

### Tumor treatment

After histological confirmation of the diagnosis, 66.4% (63/95) of all patients received tumor treatment and 12.6% (12/95) of patients had no treatment (four patients [33.3%] were not able to receive radio-chemotherapy due to a KPS < 60% and eight patients [66.7%] declined further treatment). In 20 patients (21%), adjuvant treatment was recommended, but no information was available if the treatment was indeed conducted because they were lost to follow-up. These patients were excluded from further analysis. Tumor treatment was performed following the Stupp protocol [19] in 84.1% (53/63) of the patients, 34% (18/53) of whom completed the regimen. In 15.9% (10/63), different treatment regimens were conducted (five patients received radiotherapy alone, three had chemotherapy with lomustine [CCNU], and two patients were treated with a lomustine-temozolomide combination according to the CeTeG protocol [11]).

### Treatment response and survival analysis

**Fig. 2:**
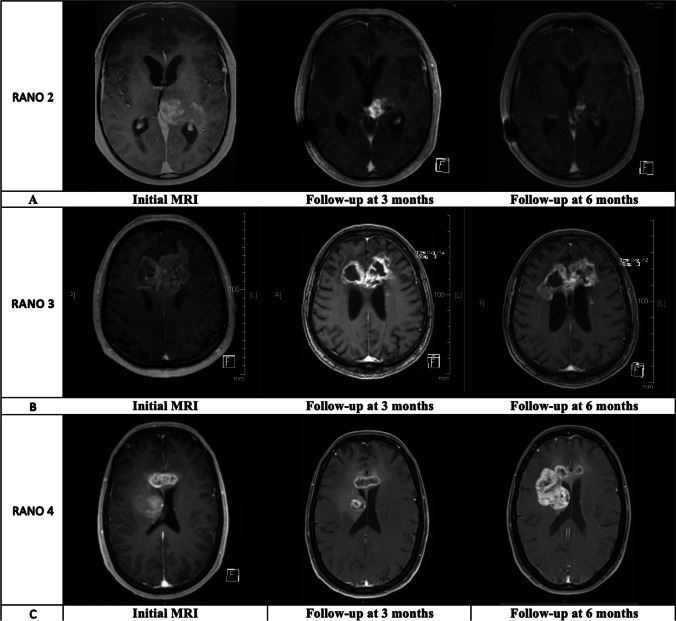
Examples of good and poor response to tumor treatment as seen on contrast-enhanced MRI and based on the RANO (response assessment in neuro-oncology) criteria, showing varying responses to treatment according to the RANO criteria comparing initial MRI with contrast with radiological response at follow-up at 3 and 6 months after tumor treatment: **A** partial radiological response (RANO 2), **B** stable disease (RANO 3), and **C** disease progression as defined by RANO 4

**Fig. 3:**
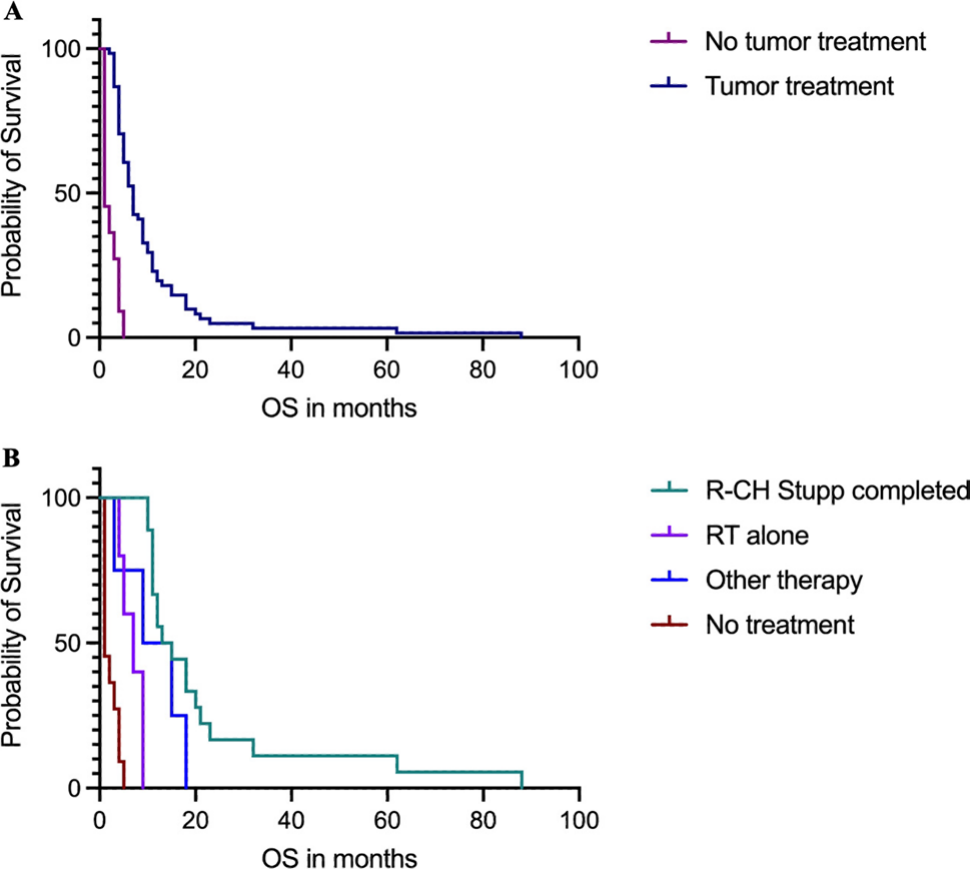
**A** Kaplan–Meier curves showing significantly longer OS in the patient group that received tumor treatment compared to the patient group without tumor treatment (log-rank test, *p* < 0.0001). **B** Kaplan–Meier curves showing the longest OS within the group that received R-CH according to the Stupp protocol, followed by other treatment protocols including CeTeG or metronomic temozolomide and RT alone, with the shortest OS in the group without tumor treatment (log-rank test, *p* < 0.0001)

Follow-up radiological data for evaluation of treatment response was available in 24 patients (25.3%), 15 of whom (62.6%) received adjuvant treatment according to the Stupp protocol, four patients (16.7%) had radiotherapy alone, three (12.5%) received chemotherapy with lomustine, one was treated following the CeTeG protocol (4.1%), and one patient (4.1%) had no treatment. At 6-month follow-up, partial radiological response (RANO 2) was found in seven patients (29.2%), four (16.7%) experienced stable disease (RANO 3), and 13 patients (54.1%) showed progression according to the definition of RANO 4. Examples of the different treatment response according to the RANO criteria are presented in Fig. 2. Median OS in the entire patient cohort was 5.5 months (95% CI 4–7), whereas 44% of the patients had an OS longer than 6 months, and only 15.8% lived longer than 12 months. When stratifying for adjuvant treatment, a significantly higher OS was detected in the patient group, who received treatment (median 7 months, 95% CI 5–9) compared to the patient group without treatment (median 1 months, 95% CI 1–4), Kaplan–Meier curves, log-rank test, *p* < 0.0001 (Fig. 3). Figure 3 demonstrates a comparison of OS with different treatment regimens, showing the longest OS for the group who completed radio-chemotherapy according to the Stupp protocol, followed by other treatment protocols including CeTeG or metronomic temozolomide, and radiation-only and the shortest OS in the group without any adjuvant treatment (log-rank test, *p* < 0.0001). In the correlation analysis, longer OS was associated with younger age (*r* =  − 0.3001, *p* = 0.01), presence of IDH mutation (*r* = 0.7584, *p* < 0.0001), adjuvant treatment (*r* = 0.2392, *p* = 0.04), and completed radio-chemotherapy according to the Stupp protocol (*r* = 0.5795, *p* < 0.0001). Neither the initial tumor volume, nor tumor location, nor the presence of methylation did show a correlation with OS in our study population with biopsied GBM (Table 2). In a multivariate analysis including age, IDH mutation, and completed radio-chemotherapy according to the Stupp protocol, only the presence of IDH mutation (*p* < 0.0001) and a completed radio-chemotherapy according to the Stupp protocol (*p* = 0.01) remained significant independent predictors of longer OS (Table 2).

**Table 2:** Analysis of parameters associated with prolonged OS

Univariate correlation analysis of parameters associated with longer OS				
**Variables**	**Correlation coefficient**	**95% CI**	***R* squared**	***p*-value**
Age	− 0.3001	− 0.4972 to − 0.0735	0.09003	0.01*
KPS	0.008342	− 0.1512 to 0.3091	0.00695	0.48
Tumor volume	− 0.03797	− 0.2956 to 0.2248	0.00144	0.77
Multifocal tumor	− 0.09319	− 0.3432 to 0.1692	0.00868	0.48
Tumor within the motor cortex	0.06643	− 0.1952 to 0.3192	0.00441	0.62
Tumor within the basal ganglia	0.1191	− 0.1436 to 0.3661	0.01419	0.37
Tumor within the corpus callosum	0.0195	− 0.2400 to 0.2765	0.00038	0.88
MGMT methylation	0.1111	− 0.2662 to 0.4589	0.01235	0.56
IDH mutation	0.7584	0.5316 to 0.8837	0.5752	< 0.0001*
Tumor treatment	0.2392	0.0079 to 0.4461	0.05722	0.04*
Completed Stupp protocol	0.5795	0.3989 to 0.7169	0.3358	< 0.0001*
**Multivariate linear regression analysis of parameters independently associated with longer OS**				
**Variables**	**Estimate**	**Standard error**	**95% CI**	***p*-value**
Age	− 0.06060	0.06345	− 0.1922 to 0.0709	0.34
IDH mutation	20.63	4.177	11.97 to 29.29	< 0.0001*
Completed Stupp protocol	5.097	1.917	1.122 to 9.072	0.01*

## Discussion

The primary focus of the analysis in this study was put on the evaluation of actual survival benefit from tumor treatment in this very specific subgroup in relation to the therapy burden. Hence, our long-term aim is to avoid possibly futile, however straining, therapy in this patient group with very limited survival odds. In 18% of our consecutive GBM patient cohort, the tumor was considered inoperable due to tumor growth patterns affecting key brain structures and therefore not permitting GTR without causing unacceptable neurological deficits. To the best of our knowledge, this is the largest patient series with newly diagnosed inoperable GBM reported in the literature so far. Our study confirmed a very limited OS (1-month median OS) in patients with inoperable GBM without tumor-specific treatment. In contrast to this, the patient cohort with inoperable GBM, who received tumor treatment (radio-chemotherapy) after histological confirmation of the diagnosis by biopsy, exhibited a significantly longer OS, clearly demonstrating a survival benefit from treatment in this patient population. However, the median OS of patients with inoperable GBM was still significantly lower compared to the reported median OS of operable GBM (9 vs. 15 months), highlighting the prognostic role of GTR in GBM patients. The identification of further clinical, radiological, and molecular parameters associated with prolonged survival is of great clinical relevance to facilitate targeted treatment in patients with GBM, in whom GTR is not reasonably achievable.

### Definition of tumor operability and prognostic role of extent of resection

The criteria applied for defining inoperability are still a matter of discussion, even though a neurosurgical expert consensus concerning this definition exists. Several factors can affect the perception of tumor resectability such as the experience of the neurosurgeon as well as the technical facilities of the institution [8, 14]. In our patient series, tumor localization within eloquent regions and multifocal tumor distribution on initial MRI were the main determining criteria for inoperability. Nevertheless, advances in functional imaging over time might have had facilitated a better interpretation of tumor relation to eloquent regions. Furthermore, surgery can not only contribute to neuro-oncological benefit but can also result in better clinical condition by prevention of threatening neurological deficits. Thus, resection of a large space-occupying mass or resection of a symptomatic lesion to relieve symptoms in a primary multifocal tumor may be discussed individually. In a longitudinal study of patient-reported quality of life, GTR was not only associated with higher PFS and OS but also with higher quality of survival [17]. On the other side, there is a risk for surgery-related complications including neurological deficits with a subsequent negative impact on the patients’ clinical condition. A meticulous weighting up of operative risks and expected benefits after diagnosis is crucial in order to provide the best achievable survival advantage from surgical treatment without additional impairment of patients’ performance status. Even though several studies have shown a survival advantage through cytoreductive surgery in GBM patients, there is an ongoing controversial discussion regarding the threshold of extent of resection (EOR) ranging from 78 to 100% [14, 18]. While it is a common practice to perform GBM resection only if GTR seems achievable, some authors hold the opinion that no threshold can be set, below which tumor resection does not extend survival. They point out the need of balancing between clinically futile and clinically worthwhile extent of tumor resections rather than a strict cutoff line [4]. Furthermore, a percentage of EOR does not directly reflect the residual tumor volume, which may substantially differ dependent on the initial tumor volume. After performing a systematic review of the literature and a meta-analysis, Brown et al. reported a low to moderate evidence that GTR substantially improves OS and PFS compared to subtotal resection. Additionally, the authors reported a lower relative risk for mortality for patients with subtotal resection compared to biopsy 1 year but not 2 years after diagnosis [4]. The role of subtotal resection in patients in whom GTR is not achievable without causing neurological deficits remains unclear and should be evaluated under consideration of the quality of life. Rather than setting a cutoff value for EOR, a weighting-up of multiple individual parameters such as tumor location and residual tumor volume as well as tumor biology seems to be necessary to allow a personalized surgical treatment in GBM patients. Therefore, a trade-off between the expected survival benefit and the quality of life is necessary, since most GBM patients would rather choose better quality of life than longer life with poor quality. The same applies to tumor treatment with radio-chemotherapy. The benefit of tumor treatment in patients with inoperable GBMs has not been determined yet. Radiation and/or chemotherapy with varying regimens have been already evaluated in patient cohorts with unresectable GBMs showing a survival benefit [2, 3]. These reports are in line with our study, where tumor treatment was associated with significantly longer median OS compared to patients, without tumor therapy. However, there are possible confounders that might have impacted this finding: patients receiving radio-chemotherapy were on average younger, which per se positively influences survival. This may have resulted in a natural selection bias in our study due to patients who did not elect to be treated after a diagnosis of GBM. It is possible that these patients in the no-treatment group tended to decompensate rapidly after diagnosis had a lower KPS upfront or had other significant comorbidities that influenced their decision. Therefore, patients in the no-treatment group would experience decreased survival time due to their intrinsic worse clinical condition. Nevertheless, multivariate analysis revealed the presence of IDH mutation and completed radio-chemotherapy according to the Stupp protocol as independent prognostic factors, whereas age was not an independent predictor of survival. The identification of further tumor-specific factors influencing OS would facilitate better treatment planning and counseling of patients with inoperable tumors.

### Tumor-specific predictors of survival in patients with inoperable GBM

Over the last years, several radiological and molecular factors were evaluated for their prognostic impact on GBM patients. Different radiological tumor characteristics including initial tumor volume, growth patterns, contact to glioma stem cell zones, ventricle contact, and involvement of the corpus callosum were evaluated as prognostic factors in our study. None of the tumor-specific locations correlated directly with OS in our patient cohort. However, the most common tumor location in the no-treatment group in our study involved the CC (80%). As it is known that patients with butterfly GBM might experience severe personality changes, this specific location might have influenced the capacity to tolerate adjuvant treatment. The topic of tumor-specific locations is under discussion in the current literature as different tumor locations offer or limit GTR and consequently influence OS. Recently, the team around Müller et al. tried to grasp this topic by introducing the “expected residual tumor volume” (eRV) and the “expected resectability index” (eRI), hereby quantifying treatment decisions, resectability, and survival [13]. The authors demonstrated that the higher the (expected) residual tumor volume and the less the (expected) resectability, the shorter the expected OS. However, the authors acknowledged that different tumor locations might result from different molecular subtypes and therefore might have had a different natural course of progression. So far, four clinically relevant GBM subtypes were identified, each with a distinct molecular pattern, response to standard therapy, and natural course [23]. Until now, no association between localization and tumor subtype has been determined; and therefore, further stratification for tumor subtype and tumor treatment might have been a valuable addition. The presence of IDH mutation and MGMT promoter methylation are well-established molecular markers associated with a favorable outcome [4, 26]. In our study, only the presence of IDH mutation was independently associated with longer OS.

### Treatment response in patients with inoperable GBM

During follow-up of our cohort, we evaluated treatment response according to the RANO criteria [25]. Most patients who underwent tumor biopsy presented with disease progression within 6 months, but the patient group with tumor treatment demonstrated a prolonged PFS. To date, several studies proved a survival benefit for GBM undergoing GTR vs. GBM with subtotal resection or biopsy [1, 5–7]. However, the group of patients with biopsied GBM was mostly not stratified for tumor treatment. A study by Balaña et al. from 2007 evaluated the survival of “biopsy-only GBM” and revealed comparable results to our study. Their cohort comprised 34 patients with grade III and IV gliomas and was therefore more heterogenous than our cohort. They also found a significant survival benefit of patients undergoing biopsy followed by radio-chemotherapy treatment compared to those without any further therapy [1]. In both studies, about 60–70% of patients received tumor treatment, which is very similar to the finding of our study (66%). Interestingly, all treatment regimens led to prolonged OS compared to the patients without further treatment. The reasons why some patients did not receive any tumor treatment were a reduced KPS, death before therapy initiation, and due to patients’ or their family’s refusal [1]. While individualized therapy concepts are of increasing importance and advancements are made regarding biomarkers and more sophisticated therapies in GBM, most treatment strategies are still based on KPS, age, and MGMT promoter status. At present, there are multiple studies that attempt to find individualized therapies according to the molecular subtype [24] that hold the potential for future effective GBM therapy.

### Limitations of the study

There are several limitations due to the retrospective nature of the study that need to be acknowledged. Due to missing data on molecular markers in the patients biopsied before 2016, our study could not provide a more diverse evaluation of the molecular markers influencing OS. Also, complete radiological data were only available in 24 of 95 patients. Therefore, the results have to be interpreted with caution due to the small sample size. Furthermore, no data on quality of life was available, hence preventing an evaluation of the compromise of quality of life during radio-chemotherapy, which is a relevant issue and represents a limitation of the study.

## Conclusion

Patients with inoperable GBM undergoing biopsy indeed experience a very limited OS. Tumor treatment is associated with significantly longer OS compared to patients not receiving treatment and should be considered, especially in younger patients with good clinical condition at presentation.

## Data Availability

All available data is already presented in the manuscript.
